# Measurement of the Activity of Wildtype and Disease-Causing ALPK1 Mutants in Transfected Cells With a 96-Well Format NF-κB/AP-1 Reporter Assay

**DOI:** 10.21769/BioProtoc.5113

**Published:** 2024-11-20

**Authors:** Tom Snelling

**Affiliations:** MRC Protein Phosphorylation and Ubiquitylation Unit, School of Life Sciences, University of Dundee, Scotland, UK

**Keywords:** ALPK1, ADP-heptose, ROSAH, Spiradenoma, Transcriptional reporter, Transfection, NF-κB, AP-1

## Abstract

Alpha-protein kinase 1 (ALPK1) is normally activated by bacterial ADP-heptose as part of the innate immune response, leading to the initiation of downstream signalling events that culminate in the activation of transcription factors such as NF-κB and AP-1. In contrast, disease-causing mutations in ALPK1 that cause ROSAH syndrome or spiradenoma allow ALPK1 to be activated in cells in the absence of bacterial infection (i.e., without ADP-heptose). This protocol describes a semi-quantitative reporter assay based on ALPK1 knockout HEK-Blue cells that measures the activity of transfected wildtype and disease-causing forms of ALPK1 by virtue of their ability to activate the transcription factors NF-κB and AP-1. These cells express a synthetic gene encoding alkaline phosphatase under the control of an NF-κB/AP-1-dependent promoter, and consequently, the activation of ALPK1 leads to the production of alkaline phosphatase, which is secreted into the culture media and can be measured colorimetrically at 645 nm after the addition of a detection reagent.

Key features

• Highly sensitive reporter assay allowing detection of low-level activity arising from ALPK1 mutants

• Optimised in 96-well plate format, requiring only 60,000 transfected ALPK1 KO HEK-Blue cells per well

• Rapid experimental design, taking only four days from start to finish

• Suitable for screening ALPK1 variants of unknown significance in an arrayed 96-well format

## Background

Alpha-protein kinase 1 (ALPK1) is an atypical protein kinase that is activated allosterically by the binding of a bacterial nucleotide sugar, ADP-heptose, to its N-terminal ADP-heptose binding domain [1]. This enables ALPK1 to phosphorylate TIFA (TRAF-interacting protein with forkhead-associated domain) at Thr9, triggering its polymerization and the consequent activation of downstream signalling events that lead to activation of transcription factors such as NF-κB and AP-1 [1,2].

ROSAH syndrome (retinal dystrophy, optic nerve edema, splenomegaly, anhidrosis, and migraine headache) is an autosomal dominant genetic disorder caused by specific mutations in ALPK1 [3]. Most of the cases of ROSAH syndrome reported so far involve the mutation of Thr237 to Met, but cases have also been identified to be caused by the mutation of Tyr254 to Cys or Ser277 to Phe [4,5]. Thr237 directly interacts with ADP-heptose, whereas Tyr254 and Ser277 are outside of the ADP-heptose binding site itself but interact with each other through a hydrogen bond [5]. In contrast, ALPK1[Val1092Ala] is a driver mutation of a rare type of hair follicle tumour called spiradenoma, which can transform into an invariably fatal form known as spiradenocarcinoma [6].

The overexpression of these disease-causing ALPK1 mutants in ALPK1 knockout (KO) cells leads to the activation of NF-κB/AP-1-dependent gene transcription in the absence of ADP-heptose by a mechanism that is dependent on an intact ADP-heptose binding site [5,7]. This conundrum was resolved when it was found that these disease-causing ALPK1 mutants are activated by mammalian nucleotide sugars such as UDP-mannose and ADP-ribose, in addition to ADP-heptose [5,7]. In contrast, the normal form of ALPK1 is specifically activated by ADP-heptose.

This protocol outlines a cell-based assay designed to measure the activation of NF-κB and AP-1-dependent gene transcription by transfected ALPK1 mutants as utilised in previous studies on this topic [5,7]. The assay uses ALPK1 KO HEK-Blue cells, which express a synthetic gene encoding a secreted form of alkaline phosphatase under the control of an NF-κB/AP-1-dependent promoter. When these cells are transfected with plasmids encoding either wildtype or mutant forms of ALPK1, their ability to activate gene transcription in the presence or absence of ADP-heptose can be quantified by measuring the levels of alkaline phosphatase in the culture medium. This is achieved through a simple colorimetric assay where the absorbance is measured at 645 nm after the addition of a detection reagent, providing an efficient method for assessing ALPK1 activity. These reporter cells have been used previously to screen for activators or inhibitors of immune pathways [8], but here we expand their utility by showing their successful use to study variants of unknown significance.

The overexpression of ALPK1 mutants in this assay enables the detection of low-level activity, which can be difficult to observe when mutants are instead expressed under their endogenous promoter in immune cells and the resulting production of cytokines or chemokines is subsequently measured. Moreover, generating complex cell models such as these would be impractical for the screening of multiple ALPK1 variants of unknown significance at scale, but can be completed with minimal effort in four days using the protocol described here.

## Materials and reagents

**Biological materials**

Cells should be cultured by incubation at 37 °C with 5% CO_2_ and tested regularly for mycoplasma using a MycoAlert Mycoplasma Detection Kit (Lonza, catalog number: LT07-318). The cells should be passaged once confluent at a ratio of 1:10 (v/v), and not used beyond 30 passages.

1. ALPK1 KO HEK-Blue cells (InvivoGen, #hkb-koalpk)

**Reagents**

The storage conditions for reagents are given in parentheses when not at room temperature. Commercial stock solutions are listed where possible, but many of these can also be prepared using standard methods.

1. ADP-heptose triethylammonium salt (InvivoGen, catalog number: tlrl-adph-l) (-80 °C)
*Note: Resuspend 250 μg in 694 μL of PBS to generate a 0.5 mM stock. Aliquot and store at -80 °C.*
2. Dulbecco’s modified Eagle’s medium (DMEM) (Gibco, catalog number: 11960-085) (4 °C)
3. Heat-inactivated foetal bovine serum (FBS) (Labtech, catalog number: FB-1001/500) (-80 °C)
*Note: FBS must be heat-inactivated to avoid high background alkaline phosphatase activity.*
4. 200 mM L-glutamine (Gibco, catalog number: 25030024) (-20 °C)
5. Penicillin-streptomycin 100× stock (Gibco, catalog number: 15140122) (-20 °C)
6. Opti-MEM I reduced serum medium (OptiMEM) (Gibco, catalog number: 31985062) (4 °C)
7. Lipofectamine 2000 (Thermo Fisher, catalog number: 11668019) (4 °C)
8. Trypan Blue solution (Gibco, catalog number: 11538886)
9. Quanti-BLUE solution 100× stock (InvivoGen, catalog number: rep-qbs) (-20 °C)
*Note: Dilute the provided solutions together to 1× in water, prepare 20 mL aliquots, and store at -20 °C.*
10. 1 M magnesium chloride (Sigma-Aldrich, catalog number: M8266)
11. Phosphate-buffered saline (PBS) (Gibco, catalog number: 10010023) (4 °C)
12. Trypsin-EDTA solution (Gibco, catalog number: 25200056) (4 °C)
13. SDS sample buffer 4× stock (Millipore, catalog number: 70607) (4 °C)
14. Benzonase endonuclease (Millipore, catalog number: E1014) (-20 °C)
15. Complete EDTA-free protease inhibitor cocktail (Roche, catalog number: 11873580001) (4 °C)
*Note: Dissolve a tablet in 1 ml of water to generate a 50× stock. Aliquot and store at -20 °C.*

**Antibodies**

1. FLAG antibody (Sigma-Aldrich, catalog number: F3165), store at -20 °C
2. GAPDH (Cell Signalling Technology, catalog number: 2118), store at -20 °C

**Plasmids**

These plasmids encode WT and mutant forms of FLAG-ALPK1 under the control of a CMV promoter and are available to request via the MRC PPU Reagents and Services website (https://mrcppureagents.dundee.ac.uk). They were purified using NucleoBond Xtra Midi Endotoxin-Free kits (Macherey-Nagel, catalog number: 740420), resuspended to 0.5 mg/mL in endotoxin-free water and stored at -20 °C.

1. pcDNA5-FRT-TO-FLAG (empty vector, denoted “EV”) (catalog number: DU41457)
2. pcDNA5-FRT/TO-FLAG-ALPK1 (catalog number: DU65668)
3. pcDNA5-FRT-TO-FLAG-ALPK1[T237M] (catalog number: DU65723)
4. pcDNA5-FRT-TO-FLAG-ALPK1[Y254C] (catalog number: DU71685)
5. pcDNA5-FRT-TO-FLAG-ALPK1[S277F] (catalog number: DU71952)
6. pcDNA5-FRT-TO-FLAG-ALPK1[V1092A] (catalog number: DU65703)
7. pcDNA5-FRT-TO-FLAG-ALPK1[R150A] (catalog number: DU71740)
8. pcDNA5-FRT-TO-FLAG-ALPK1[R150A/T237M] (catalog number: DU71743)
9. pcDNA5-FRT-TO-FLAG-ALPK1[R150A/Y254C] (catalog number: DU71741)
10. pcDNA5-FRT-TO-FLAG-ALPK1[R150A/S277F] (catalog number: DU71954)
11. pcDNA5-FRT-TO-FLAG-ALPK1[R150A/V1092A] (catalog number: DU71742)

**Solutions**

*Note: Storage conditions are given in parentheses.*

1. Antibiotic-free culture media (4 °C) (see Recipes)
2. Culture media (4 °C) (see Recipes)
3. SDS lysis buffer (use immediately) (see Recipes)

**Recipes**

1. **Antibiotic-free culture media (1 bottle)**
  **Table:** 

Reagent	Final concentration	Quantity to add
DMEM	Not applicable	500 mL
FBS	10% (v/v)	50 mL
200 mM L-Glutamine	2 mM	5.6 mL
2. **Culture media (1 bottle)**
  **Table:** 

Reagent	Final concentration	Quantity to add
DMEM	Not applicable	500 mL
FBS	10% (v/v)	50 mL
200 mM L-Glutamine	2 mM	5.6 mL
Penicillin-streptomycin 100×	1×	5.6 mL
3. **SDS lysis buffer (5 mL)**
  **Table:** 

Reagent	Final concentration	Quantity to add
SDS sample buffer 4×	1×	1.25 mL
Benzonase endonuclease	0.2% (v/v)	10 μL
1 M magnesium chloride	1 mM	5 μL
Protease inhibitor cocktail 50×	1×	100 μL
Water	Not applicable	3.635 mL

**Laboratory supplies**

Similar products can also be used, but those marked by an asterisk are highly recommended.

1. 10 cm Nunc cell culture dishes (Thermo Fisher, catalog number: 150318)*
2. 96-well Nunc cell culture plates (Thermo Fisher, catalog number: 167008)*
3. 25 mL sterile reagent reservoir (Thermo Fisher, catalog number: 11405758)
4. Clear sealing tape for 96-well plates (Thermo Fisher, catalog number: 10105383)
5. 50 mL conical centrifuge tubes (Greiner, catalog number: 227261)
6. 15 mL conical centrifuge tubes (Greiner, catalog number: 188271)
7. Serological pipettes (Thermo Fisher, catalog number: 10710810)
8. Safe-Lock 1.5 mL microcentrifuge tubes (Eppendorf, catalog number: 30123611)
9. Cellometer counting chambers (Nexcelom, catalog number: 11522186)
10. Combitips advanced 0.5 mL (Eppendorf, catalog number: 30089421)

## Equipment

Similar equipment from other suppliers can also be used instead of those listed below. Those that have been discontinued do not have a catalog number indicated.

1. Cellometer Auto 2000 (Nexcelom Bioscience)
2. Multipette M4 (Eppendorf, catalog number: 4982000012)
3. 8-channel adaptor (Integra Biosciences, catalog number: 10023451)
4. 8-channel pipettor 20–200 μL (VWR, catalog number: 89079-948)
5. Pipetman 4-Pipette Kit (Gilson, catalog number: F167360)
6. Stripettor Ultra pipet controller (Corning, catalog number: 4099)
7. CB150 cell culture incubator (Binder)
8. BioMAT 2-SF cell culture hood (Conditioned Air Solutions)
9. Allegra X-12 benchtop centrifuge (Beckman Coulter, catalog number: 392474)
10. QBT2 dry block heater (Grant)
11. Epoch plate reader (BioTek)

## Procedure

All steps are performed in a sterile culture hood, and the cells are cultured at 37 °C with 5% CO_2_. Before starting the procedure, a confluent 10 cm dish of ALPK1 KO HEK-Blue cells in culture media is required, sufficient for 2 × 96-well plates.

- **Prepare complexes of lipofectamine 2000 and plasmid in a 96-well culture plate (Day 1)**
  Dilute lipofectamine 2000 and add to the required wells:
  1. Calculate the number of wells to be transfected plus 10% extra (for 88 wells, prepare 96 wells).
  2. Each well requires 0.5 μL of lipofectamine 2000 in 24.5 μL of OptiMEM. Prepare a mastermix for 96 wells by diluting 48 μL of lipofectamine 2000 in 2352 μL of OptiMEM. Invert five times to mix thoroughly.
  3. Transfer diluted lipofectamine 2000 to a reagent reservoir and use a multi-channel pipette to add 25 μL per required well within a 96-well culture plate.
  Prepare a mastermix for each plasmid and add to the relevant wells:
  1. Calculate the number of wells to be transfected with each plasmid plus 20% extra. Each plasmid should be assayed in quadruplicate, with and without ADP-heptose. Leave an empty column for a media-only control in the 96-well plate.
  2. Each well requires 0.2 μg of plasmid in 25 μL of OptiMEM. For a 10-well mastermix of plasmid encoding WT ALPK1 (i.e. sufficient for the required 8 wells, plus 20% extra), add 2 μg of this plasmid to 250 μL of OptiMEM. Invert five times to mix thoroughly.
  3. Add 25 μL of each diluted plasmid to relevant wells of the 96-well culture plate using a repeat pipettor, remembering to change tips for different plasmids.
  4. Leave the culture plate undisturbed for at least 20 min, but no more than 6 h, to allow the formation of lipid-DNA complexes.
*Note: Prepare the cell suspension below in the meantime, which should take approximately 20 min.*
- **Add cell suspension directly into the wells of the 96-well plate containing lipid-DNA complexes (Day 1)**
  1. Aspirate the culture medium from a confluent 10 cm dish of ALPK1 KO cells and replace with 10 mL of PBS using a serological pipette.
  2. Aspirate the PBS and add 2 mL of trypsin-EDTA solution. Return the dish to the incubator until the cells have detached, which takes typically 2–3 min.
*Note: If trypsinization is taking significantly longer than this, ensure that all residual culture medium and PBS are removed during the aspiration steps since FBS present in the culture media can inhibit trypsin activity.*
  3. Add 10 mL of antibiotic-free culture media and pipette up and down with a serological pipette until a single-cell suspension has been produced. Transfer this cell suspension to a 15 mL canonical tube.
  4. Mix 20 μL of cell suspension with 20 μL of trypan blue solution and count the number of cells using standard methods, ensuring that cell viability is at least 90%.
  5. Dilute the cell suspension to 600,000 cells/mL in antibiotic-free culture media, ensuring that the total volume of the diluted cell suspension is at least 10% more than the total volume needed for the experiment.
  6. Add 100 μL per required well (i.e., 60,000 cells) into the wells of the 96-well plate that contain lipid-DNA complexes using a reagent reservoir and multi-channel pipette (see Figure 1A for expected density).
    **Figure 1.**
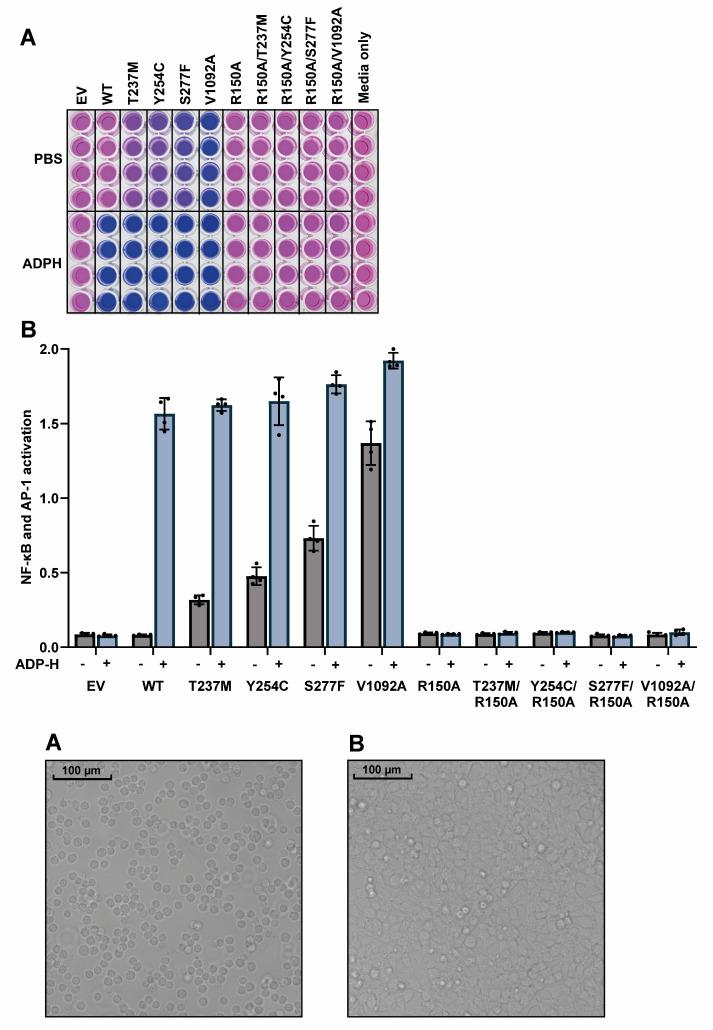
Appearance of ALPK1 KO HEK-Blue cells. Density of ALPK1 KO HEK-Blue cells 1 h after plating into 96-well plates (A) and 24 h post-transfection, prior to stimulation (B).
  7. Return the cells to the 37 °C incubator (5% CO_2_) for 24 h.
- **Replacement of culture media and stimulation of relevant wells with ADP-heptose (Day 2)**
  Prepare a mastermix of culture media containing either PBS or 5 μM ADP-heptose:
  1. At this stage, cells should be confluent (see Figure 1B). Each well requires 75 μL of culture media containing either 5 μM ADP-heptose or an equivalent volume of PBS.
  2. For PBS control wells, dilute 36 μL of PBS in 3600 μL of culture media.
  3. For ADP-heptose wells, dilute 36 μL of 0.5 mM ADP-heptose in 3600 μL of culture media.
  Aspirate wells and add the culture media:
  1. After 24 h of transfection, carefully aspirate the existing culture media using an 8-channel adaptor, being careful not to disturb the cells.
  2. Replace with 75 μL of culture media (with antibiotics) containing either PBS or ADP-heptose according to the experimental plan and return cells to the incubator for 24 h.
  3. As a control for the absorbance of the culture media itself, transfer 75 μL of the fresh culture media to an empty column.
- **Quantification of alkaline phosphatase activity in the culture media (Day 3)**
  Collection of culture media and lysis of cells:
  1. After 24 h of stimulation, transfer 50 μL of culture media from each well into a new 96-well plate using a multi-channel pipette, changing tips each time.
  2. Carefully aspirate the remaining culture media using an 8-channel adaptor, being careful not to disturb the cells. Remove residual media by centrifuging the culture plate upside down on a stack of tissues for 30 s at 500× *g*.
  3. To each well, add 30 μL of SDS lysis buffer, seal with plastic, and discard the plate lid.
  4. Incubate the plate at 75 °C for 10 min by placing it on a heat block.
  5. Analyse the unstimulated samples in duplicate (i.e., 2 of the 4 technical replicates) by SDS-PAGE followed by immunoblotting with anti-GAPDH and anti-FLAG antibodies using standard methods.
*Note: This step is essential as it will indicate any variability in the levels of expression of the different ALPK1 variants as well as the similarity in the expression levels between technical replicates.*
  Detection of absorbance at 645 nm:
  1. Add 150 μL of Quanti-Blue 1× solution to each well containing 50 μL of culture media (including the control column with fresh culture media), changing tips each time.
  2. Incubate at room temperature and take absorbance readings at 645 nm every 15 min until an optimal signal-to-noise ratio is achieved, typically 1 h (Figure 2A).
    **Figure 2.**
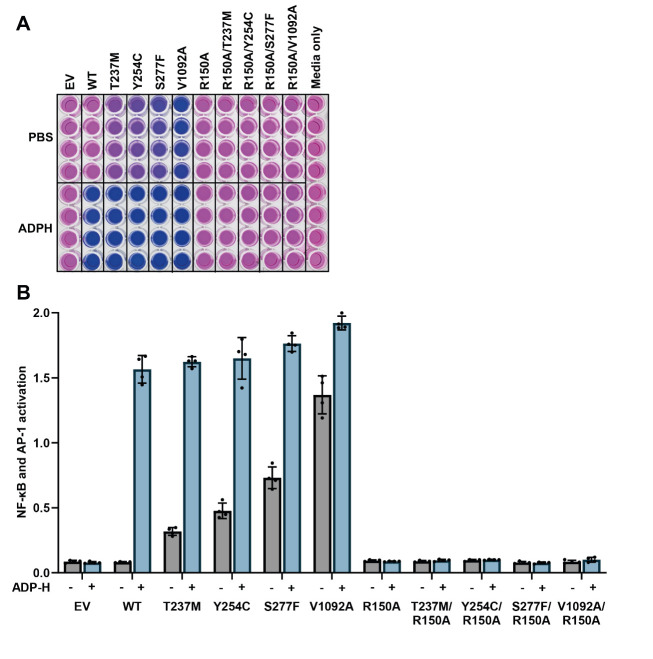
Disease-causing ALPK1 mutants have activity in the absence of ADP-heptose, which is dependent on an intact ADP-heptose binding site. (A) Appearance of the reporter plate after incubation of the culture media for 1 h with Quanti-Blue solution to detect alkaline phosphatase activity. (B) Absorbance values at 645 nm were plotted, with background absorbance from culture media alone subtracted from all other values. The bar heights represent the mean values and the error bars indicate plus and minus one standard deviation.

## Data analysis

Two alternative approaches can be used for data analysis. In both cases, the bar heights should represent the mean values, with error bars indicating plus and minus one standard deviation.

In the first approach, the average absorbance values from the culture medium-only control column are subtracted from all other values, and the resulting data are plotted (see Figure 2B). The resulting y-axis is the relative activation of NF-κB/AP-1, with the culture medium-only control set to zero since no cells were plated. In the second approach, the culture medium-only control can be excluded, and the average absorbance values from the empty vector-only controls can instead be subtracted from the values obtained from conditions where ALPK1 was expressed. This approach removes basal NF-κB/AP-1-dependent gene transcription that is independent of ALPK1, and the y-axis is, therefore, the relative ALPK1 activity.

## Validation of protocol

This protocol or parts of it has been used and validated in the following research article(s):

- Snelling, T et al (2024). Discovery and Functional analysis of a novel *ALPK1* variant in ROSAH syndrome. *bioRxiv* (Figure 2, panels A–C; Figure 4, panels B–C)
- Snelling, T et al (2023). ALPK1 mutants causing ROSAH syndrome or Spiradenoma are activated by human nucleotide sugars. *Proc Natl Acad Sci USA* (Figure 2, panel C; Figure 3, panels A–C)

## General notes and troubleshooting

**Troubleshooting**

- **Low or undetectable levels of NF-κB/AP-1 dependent gene transcription**
  This is most likely caused by poor transfection efficiency or low-quality plasmid DNA. To confirm this, check the percentage of GFP-positive cells by flow cytometry/microscopy 24 h after transfection with a GFP plasmid such as pcDNA5-FRT/TO-GFP-ALPK1 (MRC PPU Reagents and Services, catalog number: DU78380).
  Below are suggestions for troubleshooting:
  1. Ensure that 10 cm dishes of cells are not overconfluent prior to plating into 96-well plates, as this can reduce the transfection efficiency and expression of the ALPK1 constructs.
  2. Ensure that cells are evenly distributed after plating into 96-well plates and are not clumped together.
  3. Confirm that cells are free from mycoplasma and other contaminants by routine testing.
  4. Thaw a new vial of cells and ensure that they are passaged at least twice prior to transfection experiments.
  5. Check DNA purity by measuring the ratio of absorbance at 260 and 280 nm using a spectrophotometer, which should be between 1.8 and 2.0. In addition, confirm DNA integrity by agarose gel electrophoresis.
  6. Confirm that cells still express the gene encoding alkaline phosphatase by stimulation with 10 ng/mL human IL-1β (InvivoGen, catalog number: rcyec-hil1b) and measuring alkaline phosphatase activity in the culture media after 24 h.
- **Cell death during the transfection procedure**
  1. Antibiotics should be excluded from the culture media during the process of transfection, which is observed to minimise cell toxicity in this assay.
  2. Ensure that cells are plated at the required density to be confluent 24 h later, as it is observed that cell toxicity from transfection is greater at lower cell densities.
- **High background NF-κB/AP-1 dependent gene transcription**
  To determine the source of a high background signal, perform control experiments comparing alkaline phosphatase activity in the culture media of cells transfected with empty vector compared to lipofectamine alone (i.e., without DNA) and in fresh culture media with and without FBS (i.e., no cells).
  1. Ensure that FBS is heat-inactivated to denature any alkaline phosphatases that are present. It may be necessary to test multiple batches of FBS from different suppliers to minimise the background signal.
  2. Confirm that cells are free from mycoplasma and other contaminants by routine testing.
  3. Thaw a new vial of cells and passage at least twice prior to performing experiments.
  4. Ensure that plasmids are not contaminated with bacterial components by using endotoxin-free kits.
- **Cell detachment during aspiration and wash steps**
  HEK293 cells are weakly adherent and therefore easily detached during aspiration and addition steps.
  1. Do not touch the bottom of the wells when aspirating or adding culture media.
  2. Use gel-loading tips intended for SDS-PAGE on the end of an 8-channel adaptor to minimise cell detachment during aspiration steps.
  3. When adding media, pipette slowly against the side of each well to minimise cell detachment.
